# Functional outcome after interdisciplinary, acute rehabilitation in COVID-19 patients: a retrospective study

**DOI:** 10.1007/s00406-024-01862-4

**Published:** 2024-07-16

**Authors:** Nancy Elmer, Anett Reißhauer, Katharina Brehm, Daniel Drebinger, Stefan J. Schaller, Christine Schwedtke, Max E. Liebl

**Affiliations:** 1 Department of Physical Medicine, Charité – Universitätsmedizin Berlin, corporate member of Freie Universität Berlin and Humboldt- Universität zu Berlin, Charitéplatz 1, 10117 Berlin, Germany; 2 Department of Anesthesiology and Operative Intensive Care Medicine (CVK/CCM), Charité – Universitätsmedizin Berlin, corporate member of Freie Universität Berlin and Humboldt- Universität zu Berlin, Charitéplatz 1, 10117 Berlin, Germany; 3 School of Medicine, Department of Anesthesiology and Intensive Care, Technical University of Munich, Ismaningerstr. 22, 81675 Munich, Germany

**Keywords:** COVID-19, Long COVID, Acute rehabilitation, Early rehabilitation, PICS

## Abstract

**Background:**

Survivors of severe COVID-19 often exhibit a variety of sequelae including loss of mobility and ADL (activities of daily living) capacity. Acute rehabilitation (AR) is an interdisciplinary rehabilitation intervention applied early while still in a hospital setting. The goal of AR is to improve functional limitations and to increase functional independence at discharge. It is established in the treatment of patients with other severe diseases such as sepsis, polytrauma, or stroke. Data concerning AR in COVID-19 are sparse.

**Aim:**

To evaluate the changes in physical function during AR in patients after severe COVID-19.

**Methods:**

This monocentric, retrospective observational study examined the functional outcomes of a sample of COVID-19-patients who received interdisciplinary AR at a university hospital. Inclusion criteria were a positive SARS-CoV-2 test in 05/2020–01/2022 and transfer to AR after intensive care treatment. 87 patients were elegible for evaluation, 3 of whom were excluded because of death during AR. Data were extracted from the hospital information system. In a pre-post analysis, mobility (Charité Mobility Index), ADL (Barthel Index), and oxygen demand were assessed. In addition, discharge location after AR, factors associated with AR unit length of stay, and functional improvements were analyzed.

**Results:**

Data of 84 patients were analyzed. Mobility increased significantly from a median of 4 [1.25-6] CHARMI points at admission to a median of 9 [8.25-9] at discharge (*p* < 0.001). ADL increased significantly from a median of 52.5 [35.0-68.75] Barthel Index points at admission to a median of 92.5 [85–95] at discharge (*p* < 0.001). Oxygen demand decreased from 80.7 to 30.5% of patients. The majority (55.9%) of patients were discharged home, while 36.9% received direct follow-up rehabilitation. Older age correlated significantly with lower scores on the discharge assessment for mobility (Spearman’s ϱ = -0.285, *p* = 0.009) and ADL (Spearman’s ϱ = -0.297, *p* = 0.006).

**Conclusion:**

Acute rehabilitation is a viable option for COVID-19 patients with severe functional deficits after ICU treatment to achieve functional progress in mobility and ADL, reduce oxygen requirements and enable follow-up rehabilitation.

**Trial registration number and date of registration for prospectively registered trials:**

Trial registration number: DRKS00025239. Date of registration: 08 Sep 2021.

## Background

SARS-CoV-2 infection can damage numerous organ systems of the human body, with pulmonary, cardiovascular, neurological, hepatobiliary, and urogenital sequelae, among others [1]. In addition to direct organ damage and indirect organ damage like thromboembolic events, critically ill COVID-19 patients exhibit the typical complications of long-term intensive care therapy, summarized as Post Intensive Care Syndrome (PICS) [2]. The term PICS is used to describe the presence of any impairment affecting the physical, psychiatric, or cognitive domains as a result of critical illness [3]. Survivors of critical illness related to COVID-19 are at high risk of developing PICS for a number of reasons [3]. The exact prevalence of PICS is unknown; however, Marra et al. followed 406 ICU survivors at 3 and 12 months and found at least one deficit in a PICS domain in 64% and 56% of patients, respectively [4].

Severe acute respiratory distress syndrome in the context of a SARS-CoV-2 infection may have long-lasting or persisting symptoms after ICU discharge, with a significant overlap with PICS (shortness of breath, brain fog, fatigue, chronic pain, motor or sensory dysfunction), and may exacerbate its symptoms [5]. PICS after COVID-19 includes not only new or worsening impairments (anxiety, post traumatic distress, sarcopenia, muscle wasting) but also the specific sequelae after COVID-19 disease (heart palpitations, cough, headache, autonomic dysfunction), while the post COVID-19 condition (PCC), in contrast, is frequently found after non-hospitalized courses of infection, and regardless of the severity of the infection [6].

Possible mechanisms contributing to the pathophysiology of such a severe COVID-19 course of disease include virus-specific pathophysiologic changes, immunologic abnormalities and inflammatory damage in response to acute infection, as well as functional sequelae, and sequelae of therapy [7, 8]. The latter might have been unintended or calculatedly accepted in order to save the patient’s life, specifically complications of prone positioning [9].

Acute rehabilitation (AR), also known as very early rehabilitation, is located in the hospital [10]. To counteract impending complications of immobility and to influence the course of recovery as favorably as possible, acute rehabilitation is integrated as early as possible in the treatment course of these patients during the acute treatment phase [11]. AR is characterized by its multi-professional rehabilitation team and its functioning-oriented approach. Particularly in COVID-19 patients, AR must take into account a number of sequelae in addition to concomitant diseases (e.g., impaired lung function requiring oxygen therapy, secondary complications) [12]. Elmer et al. described factors with relevance to acute rehabilitation in these patients, such as “silent hypoxia” (desaturation without subjective dyspnea) and complications caused by prone positioning, such as pressure ulcers of the ventral body parts as well as peripheral nerve lesions [13–15].

In sepsis patients, AR is associated with an advantage in short-term and long-term survival [16]. Data concerning AR in COVID-19 are sparse. In a prospective study by Rodrigues et al. in 42 patients after severe COVID-19 and intensive care treatment followed by inpatient rehabilitation in a rehabilitation clinic, there was a significant improvement in muscle strength, balance, physical performance and activities of daily living (ADL) ability as well as fatigue from admission to discharge [17]. A cohort study by Udina et al. demonstrated a significant effect of multidisciplinary therapies (rehabilitation in a post-acute care facility) in COVID-19 patients (with and without intensive care unit stay) on ADL (Barthel Index) and physical performance. Assessments were carried out on days 1 and 10 of the intervention [18]. A case series by Chuang et al. confirmed the effectiveness of acute inpatient rehabilitation in COVID-19 fatigue, comparing pre-/post assessments [19].

While there is growing evidence for COVID-19 rehabilitation after hospital stays, there is only preliminary evidence AR effectiveness after severe COVID-19.

The primary aim of this retrospective study were to evaluate functional improvement of mobility, ADL and oxygen demand between admission and discharge. The secondary aim was to investigate whether AR has an impact on independence and the corresponding discharge location. Further aims were to explore which factors influence the course during AR.

## Methods

### Study design

This study analyzes the COVID-19 subgroup of a larger monocentric retrospective study on the clinical outcomes of AR from the years 2011–2020 [20]). It included patients admitted to the interdisciplinary acute rehabilitation unit (ARU), a specialized early rehabilitation ward of a university hospital. The study was approved by the ethics committee of Charité Universitätsmedizin Berlin (EA4/067/20) and registered in the German Register of Clinical Trials at the Federal Institute for Drugs and Medical Devices (BfArM) (register number DRKS00025239). Reporting was carried out in accordance with the STROBE (Strengthening the Reporting of Observational Studies in Epidemiology) Guidelines [21].

### Data collection process and database

Data were extracted by a health professional (physician) from the hospital information system (SAP i.s.h.med) and transferred into a SPSS (IBM, Chicago, Illinois, USA) database. Another researcher (physician) double-checked the data to reduce errors in data extraction and check for plausibility.

### Statistical analysis

Data were analyzed descriptively according to their distributional and scaling properties. Primary outcome variables were improvements in mobility and ADL. Wilcoxon tests were used for non-parametric significance tests; the effect sizes were calculated using Cohen’s d. Secondary outcomes were oxygen demand and ARU length of stay. Possible influences of risk factors (ICU length of stay, duration of mechanical ventilation, sex, age, and BMI) on functional outcomes and ARU length of stay were analyzed using Spearman’s correlation and Mann-Whitney U-test. All resulting p-values are exploratory. The statistical analysis was performed with IBM SPSS 28.

### Participants

All patients transferred to the ARU after treatment of COVID-19 between May 2020 and January 2022 were included in the study. All participants fulfilled the WHO definition of a severe infection: SpO₂ < 94% on room air, respiratory rate > 30/min, or lung infiltrates > 50% in the course of their illness [22]. The referral for AR was initiated by intensive care physicians and indicated by rehabilitation specialists. Premature termination of the AR due to death was an exclusion criterion, as well as missing data in baseline demographic data or functional outcome parameters.

87 patients met the inclusion criteria. 3 patients were excluded because they died during the ARU stay (2 deaths due to complications of secondary sclerosing cholangitis (SSC), 1 death due to cerebral hemorrhage). No cases had to be excluded due to missing data.

### In-patient acute rehabilitation intervention

All COVID-19 patients received physiotherapy (PT) and occupational therapy (OT) as well as speech and swallowing therapy and psychological/psychosomatic support, depending on the indication. The therapies were carried out in a fixed amount of 20 therapy units (30 min each) per week, with individually varied intensity. The basic PT program included strengthening motor skills, balance training, aerobic training, and gait training, depending on the patient’s individual abilities. Another focus laid on respiratory therapy, patients were also instructed in self-exercises. OT focused on skills training for the basic ADLs and the fitting of assistive aids. Therapy goal setting and goal attainment scaling were performed in weekly multiprofessional team meetings. Regular discharge criteria were the functional capacity to receive out of hospital, follow-up rehabilitation, alternatively the capacity for hospital discharge.

Oxygen saturation, heart rate, respiratory rate and dyspnea (Borg scale) were regularly monitored during mobilization. In case of oxygen desaturation, potential “silent hypoxia” was documented in the case of desaturation without subjective dyspnea or physiological reaction.

### Outcome measures

The following variables were collected from the included patients:

- Baseline demographic and patient-specific data: age, sex, BMI, pre-existing conditions (risk factors for severe COVID-19 progression [23]).
- Disease-related data: length of stay in intensive care, length of stay in AR, duration of ventilation, extracorporeal membrane oxygenation (ECMO) treatment, isolation (due to infectious or multi-drug resistant pathogens), relevant preexisting conditions at baseline (before admission to hospital, e.g. diabetes mellitus, lung disease, cardiovascular disease (arterial hypertension), as risk factors for severe COVID-19) and therapy-related sequelae [23].
- Functional parameters of AR: admission and discharge assessments on mobility and ADL (Charité Mobility Index (CHARMI), which measures the independent mobility level with an ordinal scale from 0 to 10, based on the International Classification of Functioning (ICF) [24]; Barthel Index (BI), which measures ADL and body functions [25]); oxygen demand (SpO₂< 94% on room air).
- Discharge location (postacute rehabilitation, discharge to home, nursing facility, other hospital department).

## Results

Data of 84 patients who underwent AR after COVID-19 were included in this study. Baseline characteristics and disease-specific patient data are shown in Table 1.

**Table 1:** Demographic and disease-specific data, relevant preexisting conditions

**Demographic and disease-specific data (** ***n*** ** = 84)**	
Age (years)	57.0 ± 12.39
Female sex	35 (41.7%)
BMI (kg/m^2^)	28.9 ± 7.76
ICU treatment^a^	78 (92.9%)
Mechanical ventilation^a^	74 (86.9%)
Duration of mechanical ventilation (days; *n* = 62)	23.4 [12.7–44.6]
ECMO treatment^a^	19 (22.6%)
Colonization/infection with MDRO requiring contact precautions	30 (35.7%)
Length of stay ICU (days; median [IQR]; *n* = 76)	32.5 [18-54.5]
Length of stay ARU (days; median [IQR])	24.5 [16-33.75]
**Preexisting conditions (with relevance for acute rehabilitation)**	
Diabetes mellitus	30 (35.7%)
Arterial hypertension	47 (56.0%)
Lung disease (unspecified)	35 (41.7%)
Obesity (BMI ≥ 30)	29 (34.5%)

Numbers are presented as mean ± standard deviation, median [inter quartile range] or n (%)

^a^ Before ARU transferral

ARU = acute rehabilitation unit; BMI = body mass index; MDRO = multidrug resistant organisms; ICU = intensive care unit; ECMO = extracorporeal membrane oxygenation

### Functional outcomes

Table 2 provides an overview of the functional outcomes.

**Table 2:** Functional outcomes

Functional Outcomes	median [IQR]	*p*-value pre/post stay in ARU
Mobility – CHARMI		
ARU admission	4 [1.25-6]	
ARU discharge	9 [8.25-9]	
Δ Mobility in ARU	4 [3–6]	*p* < 0.001
ADL – Barthel Index		
ARU admission	52.5 [35.0-68.75]	
ARU discharge	92.5 [85–95]	
Δ ADL in ARU	30 [25–50]	*p* < 0.001
Oxygen demand		
Patients with oxygen demand on admission	80.7% (67 of 83)	
Patients with oxygen demand on discharge	30.5% (25 of 82)	

ARU = acute rehabilitation unit; ADL = activities of daily living

Mobility and ADL: Patients had a median CHARMI of 4 [1.25-6] points at ARU admission, i.e. they were able to sit at the edge of bed, but unable to stand up independently. At discharge, the median CHARMI value was 9 [8.25-9] points, with 9 points corresponding to independent stair climbing. The improvement in mobility was 4 [3–6] CHARMI points and was significant (Wilcoxon-Test: *n* = 84; z = 7.987; *p* < 0.001, Cohen’s d = 2.212).

There was also a significant improvement of 30 [25–50] points in Barthel Index during the course of acute rehabilitation (Wilcoxon-Test: *n* = 84; z = -7.978; *p* < 0.001, Cohen’s d = 2.080).

With 80.7% of patients dependent on oxygen substitution on admission, oxygen demand was reduced to 30.5%, who still met the indication criteria for long-term oxygen therapy and were prescribed home long-term oxygen therapy (LTOT) therapy.

Table 3 lists the most common disease and therapy sequelae, which were functionally relevant in the course of acute rehabilitation after severe COVID-19.

**Table 3:** Frequent sequelae of severe COVID-19 with relevance for acute rehabilitation

Organ system	Sequelae	%	*n*	*n*_total_
Pulmonal	O₂-demand at ARU admission	80.7	67	83
	Silent hypoxemia	32.9	27	82
	Residual tracheostomy	38.6	32	83
Cardiovascular	Resting tachycardia	23.5	19	81
Hepatobiliary	Secondary sclerosing cholangitis	13.6	11	81
Renal	Hemodialysis	13.1		
Neuropsychiatric	Psychiatric impairments (e.g. anxiety, signs of depression or PTSD)	32.1	27	84
Neurological	Peripheral nerve lesion	11.9	10	84
Integument	Ventral pressure ulcers	52.4	44	84

ARU = acute rehabilitation unit; PTSD = post-traumatic stress disorder

### Potential factors associated with ARU length of stay and physical function

ICU length of stay (LOS) correlated significantly with ARU LOS (*n* = 76; Spearman-ϱ = 0.226, *p* = 0.049). ICU length of stay (LOS) correlated significantly with lower mobility and ADL function at the time of ARU admission (*n* = 76; CHARMI: Spearman-ϱ = -0.247, *p* = 0.032; Barthel Index: Spearman-ϱ = -0.289, *p* = 0.011). At the time of ARU discharge, no significant correlation of the functional outcomes with ICU length of stay were detectable (*n* = 76; CHARMI: Spearman-ϱ = -0.022, *p* = 0.849; BI: Spearman-ϱ = -0.094, *p* = 0.417).

Duration of mechanical, ventilation did not demonstrate any significant influence neither on the ARU length of stay, nor on the functional outcomes at ARU admission and ARU discharge (*n* = 62; ARU LOS: Spearman-ϱ = 0.065, *p* = 0.615; CHARMI admission: Spearman-ϱ = -0.096, *p* = 0.457; CHARMI discharge: Spearman-ϱ = 0.086, *p* = 0.508; BI admission: Spearman-ϱ = -0.086, *p* = 0.508; BI discharge: Spearman-ϱ = 0.108, *p* = 0.405).

Similarly, there was no significant correlation of age on ARU length of stay, mobility or ADL function at ARU admission (ARU length of lay: Spearman-ϱ = 0.129, *p* = 0.241; CHARMI admission: Spearman-ϱ = -0.143, *p* = 0.195; BI admission: Spearman-ϱ = -0.159, *p* = 0.149). However, older age correlated significantly and moderately with lower scores on the discharge assessment for mobility and ADL (CHARMI discharge: Spearman-ϱ = -0.285, *p* = 0.009; BI discharge: Spearman-ϱ = -0.297, *p* = 0.006).

There were no significant group differences between the sexes in ARU LOS, admission or discharge assessments for mobility and ADL (Mann-Whitney U-tests: *n* = 84, LOS: U = 724.00, z = -0.212, *p* = 0.225; CHARMI admission: U = 912.00, z = 0.502, *p* = 0.616; BI admission: U = 850.00, z = -0.068, *p* = 0.946; CHARMI discharge: U = 805.50, z = -0.585, *p* = 0.558; BI discharge: U = 698.00, z = -1.545, *p* = 0.122).

BMI showed no significant correlation with ARU length of stay, admission or discharge assessments in ADL or mobility. (ARU LOS: Spearman-ϱ = -0.153, *p* = 0.182; CHARMI admission: Spearman-ϱ = 0.062, *p* = 0.592; CHARMI discharge: Spearman-ϱ = -0.029, *p* = 0.800; BI admission: Spearman-ϱ = 0.102, *p* = 0.376; BI discharge: Spearman-ϱ = -0.098, *p* = 0.392).

### Discharge location

55.9% of patients could be discharged home (with or without home care) and 36.9% to postacute follow-up rehabilitation treatment (Table  4). 2.4% of patients were transferred to a nursing facility and 4.8% of patients were transferred to another hospital department after their ARU stay for further hospital treatment (1 due to reactivated tuberculosis, 1 for continuation of a previously started chemotherapy, 1 for transcatheter aortic valve implantation (TAVI), 1 for liver transplant listing).

**Table 4:** Discharge location

Discharge location	
Postacute rehabilitation	36.9%
Discharge to home (with or without homecare)	55.9%
Nursing Facility	2.4%
Transferal to other hospital department	4.8%

## Discussion

In summary, it appears that AR can lead to improvement in relevant functional domains such as mobility and ADL after severe COVID-19.

For our sample, we were able to demonstrate highly relevant functional improvements in mobility and ADL between admission and discharge (CHARMI admission 4 [1.25-6] to discharge 9 [8.25-9]) and ADL (BI admission 52.5 [35.0-68.75] to discharge 92.5 [85–95]), confirming the results of similar studies. A prospective multicenter observational study by Puchner et al. included a total of 23 individuals discharged after severe to critical COVID-19 [12]. The mean age of their study participants was 57 years, 70% were male, and they were hospitalized for 32 ± 16 days after 19 ± 4 days in the intensive care unit. After 24 ± 5 days of interdisciplinary rehabilitation, they found a significant improvement in walking distance in the 6-minute-walk-test (6MWT) from 323 ± 196 to 499 ± 103 m and in ADL function in 83 ± 18 to 97 ± 7 BI points. On the other hand, Curci et al. included 41 COVID-19 patients transferred directly from the ICU (mean ICU LOS of 17.98 ± 8.66 days), also predominantly men, but with a mean age of 72 years [23]. After a mean LOS in early rehabilitation of 32 ± 9 days, they also found a statistically significant improvement in BI (43 ± 26 to 85 ± 16) and 6MWT (240 ± 81 to 303 ± 112 m) [23]. In our study in comparison, a larger number of patients were studied, with a longer ICU stay (32.5 [18-54.5] days), and relevantly lower levels of functioning on admission (6-minute walk test was not even possible in our sample).

According to Rodrigues et al., older age and longer ICU length of stay could be identified as risk factors for worse functional outcomes. 42 patients (predominantly men, mean age 62) with a mean ICU length of stay of 20 days, among them 9.5% with ECMO treatment, were included [17]. In our study, with a slightly lower mean age of 57 ± 12.39 years, older age as well correlated significantly with lower scores in the discharge mobility and ADL.

Duration of ventilation had no significant effect on functional outcome measures in our study, but ICU LOS correlated significantly with poorer ARU admission assessments for ADL and mobility as well as longer stays in ARU, which confirms the results of Rodrigues et al. [17].

In a descriptive cohort study of 159 patients after COVID-19 inpatient treatment, 44.3% still required continued rehabilitation at 5 months after hospital discharge [26]. In our study, 36.9% of patients were transferred to follow-up rehabilitation after their ARU stay. As many rehabilitation clinics require certain levels of functioning in mobility and ADL, an important role of the AR in hospitals is to enable patients for follow-up rehabilitation. Thus, the percentage of follow-up rehabilitation can be cautiously interpreted as a criterion for AR success, especially as AR is associated with lower mortality in sepsis patients [16]. Another functional outcome measure with regard to the discharge location is the rate of patients with the need for post-hospital nursing care. We could show an extremely low rate of 2.4% here.

### Aspects of acute rehabilitation after COVID-19 that require interdisciplinary therapy

The multi-organ involvement of COVID-19 may require interdisciplinary involvement of different specialist teams also during AR. Our data illustrate this: Pulmonary symptoms are predominantly relevant to function and therapy, with 80.7% of our patients presenting with oxygen demand at ARU admission. Dyspnea is the known as the most common persistent symptom after acute COVID-19 treatment, with a prevalence of 42 and 66%, respectively, in two larger post-discharge follow-up studies [27, 28]. Survivors of severe COVID-19 have been found to have a spectrum of pulmonary manifestations ranging from mild parenchymal destruction (with or without need of chronic oxygen supplementation) to fibrotic lung injury [7]. In an American COVID-19 follow-up study, supplemental oxygen demand due to persistent hypoxemia was found in 6.6% of patients, respectively, after 60 days [29]. Although our study indicated the success of AR by a relevant absolute reduction in oxygen dependence, a remarkable share (30.5%) of our patients remained oxygen dependent at discharge. For these patients, this implies a continuing health limitation and consequently a reduced quality of life and participation. Our study shows a high oxygen demand on admission and discharge (higher than comparative studies [27, 28]), most probably due to the severe courses and early transfer from the ICU.

Furthermore, “silent hypoxemia”, i.e. oxygen desaturation without subjective symptoms of dyspnea, appeared in a remarkably high rate 32.9% of ARU patients. A case series by Elmer at al. “silent hypoxemia” describes this phenomenon in the early treatment phase after COVID-19 infection [13]. This plays an important role in rehabilitation interventions, since the usual parameters such as dyspnea, heart rate and respiratory rate cannot be used safely to control training intensity.

A cardiac manifestation requiring treatment occurred in 23.5% of our sample in form of resting tachycardia. Similar to other observational studies, we could show that palpitations and tachycardia are present in approximately 14–20% of patients after COVID-19 [30, 31]. Autopsy studies detected the virus in cardiac tissue samples of 62.5% of deceased COVID-19 patients [32].

Secondary sclerosing cholangitis (SSC) appeared in 13.6%. It is known that changes in cholestasis parameters occur in a considerable number of patients in during COVID-19 [33]. In their cross-sectional study, Hartl et al. analyzed 496 patients hospitalized due to SARS-CoV-2 infection, 15% of whom developed SSC [29], comparable with our case numbers. SSC occurs more frequently after COVID-19 than after other severe diseases, especially if there have been previous chronic conditions [34].

In our study, neuropsychiatric impairments were evident in 32.1% of patients having received psychosomatic or psychiatric co-management, confirming above figures. Individuals with COVID-19 may experience a range of psychiatric symptoms that can persist or occur even months after initial infection [35, 36]. In a cohort of 402 COVID-19 survivors in Italy, approximately 56% had pathologic findings in at least one of the domains assessed for psychiatric sequelae (post-traumatic stress disorder (PTSD), depression, anxiety, insomnia, and obsessive-compulsive symptoms) one month after hospitalization [37]. Clinically significant depression and anxiety have been reported in around 30–40% of patients after COVID-19 [1]. In our study, the number of patients with psychiatric symptoms was comparably high with 32.1%. However, no precise distinction was made between different types of psychiatric symptoms.

COVID-19 can also affect the renal system. Severe acute kidney injury (AKI) requiring renal replacement therapy occured in 5% of all hospitalized patients and in 20–31% of critically ill patients with acute COVID-19, especially in patients requiring mechanical ventilation [7, 38, 39]. Our results are well below these numbers with 13.1%, but for accurate interpretation is has to be mentioned that our numbers only include renal replacement therapy while in acute rehabilitation and not intermittent need in intensive care.

Prone positioning (“proning”) sequelae may be individually relevant in acute rehabilitation [9]. Our study demonstrated that ventral pressure ulcers (52.9%) and nerve lesions (11.3%) are particularly characteristic. However, in a retrospective study by Elmer at al. it was shown that acute rehabilitation allowed patients to be discharged at comparable mobility and ADL levels as patients without proning sequelae [14]. Patients with critical COVID-19 are also at increased risk for superinfection with bacterial, fungal (e.g. pulmonary aspergillosis), or other pathogens [40–42]. We can also confirm this in our study, as 35.7% of the sample showed colonization or infection with multidrug resistant pathogens requiring increased contact precautions, presumably associated with the prolonged intensive care treatment and multiple use of antibiotics.

The multi-organ involvement of COVID-19 demonstrate the need for an interdisciplinary approach in the AR phase [43, 44] where a team of different specialists is required for treatment. Thanks to its multidisciplinary team and its location in an acute hospital, the ARU is well equipped to treat patients with these multiple impairments.

Physical, psychological and cognitive impairments observed in post-COVID-19 ICU survivors are similar to those observed in PICS patients [6]. PICS can also develop after a severe septic infection in the intensive care unit (ICU) [4], which is also characterized by ICU-acquired weakness (ICU-AW). ICU-AW occurs in 46% of critically ill patients with sepsis, multiple organ failure or long-term ventilation [4]. PICS is a concept that describes physical and non-physical long-term sequelae after intensive care, in the four domains of mental health, cognition, physical function, and health-related quality of life [45]. Critically ill patients COVID-19 patients are considered vulnerable to developing post intensive care syndrome, as they may suffer direct and indirect organ damage, therapy- and immobility-associated sequelae as well as long-term impairments of functioning [7]. Our cohort largely fulfills these criteria. In addition to PICS, there are the specific somatic sequelae after severe COVID-19 disease. Organ-specific sequelae after COVID-19 and symptoms of PICS are difficult to separate and overlap several areas. Considering the functional improvements in our study, it could be hypothesized that AR may reduce impairment and morbidity in the context of PICS.

### Limitations

This study has some limitations. Firstly, we do not have a control group and it is not possible to report on the causality of the observed findings. Pulmonary findings such as spirometry and exact oxygen requirements were also not evaluated. There is also a lack of information on psychiatric impairments in our study, which could have been of interest in comparison to recent research [45]. Finally, we do not have an exact number of PICS in our sample, partly due to a missing clear definition of PICS [46].

## Conclusion

According to WHO recommendations, hospitals should establish specialized rehabilitation units for inpatients with complex needs [47]. Acute rehabilitation is a viable option for COVID-19 patients with severe functional deficits after ICU treatment to achieve functional progress in mobility and ADL, reduce oxygen requirements and enable follow-up rehabilitation.
